# Redistribution of branched-chain amino acid intake between active and inactive phases modulates hepatic metabolism in rats

**DOI:** 10.3389/fnut.2026.1754879

**Published:** 2026-04-09

**Authors:** Kuai Yu, Meng Lian, Hao Li, Tong Jin, Liling Xiao, Junhang Chen, Huihong Ma, Caihong Wu, Rong Hu, Heng Luo

**Affiliations:** 1 State Key Laboratory of Discovery and Utilization of Functional Components in Traditional Chinese Medicine, Guizhou Medical University, Guiyang, China; 2 Natural Products Research Center of Guizhou Province, Guiyang, China; 3 Center for Tissue Engineering and Stem Cell Research, Guizhou Medical University, Guiyang, China; 4 Translational Medicine Research Center, Guizhou Medical University, Guiyang, China; 5 Guizhou Biomanufacturing Laboratory, Guizhou Medical University, Guiyang, China; 6 Key Laboratory for Research on Autoimmune Diseases of Higher Education Schools in Guizhou Province, Guiyang, China; 7 Department of Physiology, School of Basic Medical Sciences, Guizhou Medical University, Guiyang, China

**Keywords:** branched-chain amino acids, circadian rhythm, homeostasis, liver, metabolomics, redistribution

## Abstract

**Background:**

Branched-chain amino acids (BCAAs) regulate protein metabolism and energy homeostasis; however, elevated BCAA exposure is associated with cardiometabolic risk. While overall feeding timing influences metabolic health, the metabolic consequences of circadian phase-dependent BCAA enrichment remain insufficiently defined.

**Methods:**

A total of 18 male Sprague–Dawley rats were assigned for 12 weeks to diets designed to be nominally isocaloric and isonitrogenous while redistributing BCAA content across the light–dark cycle: control (Ctrl), D + N − (BCAA-enriched during the inactive/light phase and reduced during the active/dark phase), or D − N + (BCAA-reduced during the inactive phase and enriched during the active phase). Growth trajectories, plasma lipids, liver and skeletal muscle untargeted metabolomes, pathway enrichment networks, and selected hepatic antioxidant indices were evaluated, and skeletal muscle mammalian target of rapamycin complex 1 (mTORC1) signaling was assessed by S6 phosphorylation at the terminal fasted time point.

**Results:**

Final body weight and skeletal muscle p-S6/S6 did not differ across the groups. However, D − N + showed more uniform growth trajectories (lower residual variance) compared to Ctrl and D + N−. Plasma LDL-C was higher in D + N − than Ctrl. Untargeted metabolomics demonstrated the strongest group separation in the liver, driven predominantly by lipid-related features including putative bile acid-annotated metabolites, whereas skeletal muscle changes were more heterogeneous. Tissue BCAA and detected branched-chain keto acid (BCKA) abundances were largely unchanged. Pathway–network analysis indicated that inactive-phase BCAA enrichment (D + N−) was associated with hepatic glutathione-pathway enrichment and downregulation of nutrient-handling modules (e.g., protein digestion/absorption), whereas active-phase enrichment (D − N+) showed lower enrichment of stress/immune-adjacent pathways (e.g., necroptosis/sphingolipid signaling). Consistently, hepatic total superoxide dismutase (T-SOD) activity was higher in D − N + than in D + N−, while GSH/GSSG was unchanged. In the skeletal muscle, D − N + was associated with enrichment of energy/biosynthetic support pathways (purine/nucleotide metabolism; pantothenate/coenzyme A [CoA] biosynthesis) without a sustained endpoint shift in mTORC1 readout.

**Conclusion:**

Redistributing BCAA exposure across circadian phases produces measurable, timing-dependent metabolic differences, with the liver exhibiting the most coherent response. Aligning BCAA enrichment to the active phase is associated with a more favorable hepatic antioxidant/stress-signaling profile, whereas inactive-phase enrichment coincides with a mild dyslipidemic signal and redox-adjacent pathway remodeling.

## 1 Introduction

Branched-chain amino acids (BCAAs), comprising leucine, isoleucine, and valine, are essential amino acids that play crucial roles in various metabolic processes. They are involved in protein synthesis, energy production, and neurotransmitter metabolism (1, 2). These properties have led to widespread use of BCAA supplementation in athletic and aging populations to promote muscle growth and delay catabolic processes. However, other studies also suggest that BCAAs influence insulin secretion and have been implicated in various health conditions, including diabetes, dyslipidemia, obesity, and cardiovascular disease (3, 4), and restricting their intake can reverse these adverse effects (5, 6). This paradox suggests that while BCAAs are vital for normal metabolic function, their imbalance or dysregulation can contribute to metabolic disease. This duality underscores the complex, context-dependent nature of BCAA biology and highlights the need for a deeper understanding of the factors that modulate their effects *in vivo*.

One important context for nutrient metabolism is the circadian timing of intake. Virtually all aspects of mammalian metabolism are regulated by circadian rhythms—endogenous ~24-h cycles in physiology and behavior (7). These rhythms anticipate daily light/dark cycles and coordinate metabolic processes with periods of activity and rest (8). Feeding is a potent circadian “zeitgeber,” and the timing of food intake can entrain peripheral clocks in metabolic organs, such as the liver, muscle, and adipose, to align with nutrient availability (9). When feeding patterns are synchronized with the organism’s active phase (daytime for humans and nighttime for nocturnal rodents), metabolism is optimized to efficiently process and store nutrients. By contrast, consuming food at inappropriate times, such as eating during the usual sleep/inactive phase, can cause circadian misalignment and metabolic dysregulation (9, 10). Experimental studies have shown that mistimed feeding leads to a desynchrony between central and peripheral clocks, reducing metabolic efficiency and predisposing to weight gain and adiposity (9).

Despite growing recognition that nutrient timing matters, the majority of studies to date have focused on whole-diet feeding patterns (e.g., time-restricted feeding (TRF) *vs. ad libitum* feeding) rather than the timing of specific dietary components. In the case of BCAAs, there have been only a handful of studies linking them with circadian rhythms (11–14), the majority of which only focus on sports-related contexts. However, this is beginning to change, as comprehensive reviews are now synthesizing the evidence that plasma BCAA concentrations are under tight circadian control and that disrupting this rhythm is closely associated with cardiometabolic health (2). For example, a key study showed that a single high-BCAA meal given to mice at the end of the active period (analogous to “dinner” time) caused rapid cardiac hypertrophy (e.g., increased cardiomyocyte size, mTOR activation, and biventricular weight-to-tibia length ratio) but worsened cardiac function (e.g., decreased cardiac output), whereas the same BCAA load in the early active period (“breakfast” time) had no such deleterious effect (11). The late-phase BCAA feeding exaggerated mTOR activation in the heart and accelerated heart failure progression in a disease model, an effect abolished by blocking the cardiac circadian clock. This striking finding implies that the cardiometabolic impact of BCAAs is highly time-of-day-dependent. However, beyond isolated organ-specific studies, little is known about how distributing BCAA intake across different times of day affects systemic metabolism and metabolic health indicators.

In light of evidence that both BCAA dose and circadian feeding context shape metabolic health, we designed a feeding intervention that redistributes BCAA availability across the light–dark cycle while keeping daily energy and nitrogen intake broadly comparable. We hypothesized that concentrating BCAA intake during the inactive phase (D + N−) would be associated with less favorable lipid/redox homeostasis than aligning BCAA enrichment to the active phase (D − N+), with the liver showing greater timing sensitivity than skeletal muscle. To test this, we quantified growth and plasma lipids, profiled liver and skeletal muscle metabolomes, and assessed hepatic antioxidant indices and skeletal-muscle mTORC1 signaling at a standardized end-of-active-phase sampling time point.

## 2 Materials and methods

### 2.1 Animals and diets

This experiment was approved by the Ethics Committee of Guizhou Medical University (No. 2200348). Prior to the current study, to calculate ideal feed amount, feeding and weight information was collected from other studies of our lab where Sprague–Dawley rats were subjected to 20 consecutive days of *ad libitum* access to an American Institute of Nutrition 1993 Growth Diet (AIN-93G) (standard diet), yielding data from 9 rats with body weight ranging from 67.1 g to 274.1 g and food consumption (per capita) between 3.1 g and 43.5 g. Thus, a scatter plot was made, a logarithmic curve was fitted, and its equation was derived. The equation was then used for feed calculation based on animal weight with rigorous monitoring and dynamic adjustment to target comparable intake (nominally isocaloric/isonitrogenous), but minor deviations occurred among the groups.

Three isonitrogenous diets were formulated based on the standard AIN-93G diet and manufactured by Xiao Shu You Tai (Beijing, China) Biotechnology Co., Ltd. The control (Ctrl) diet consisted of the standard AIN-93G formulation. The BCAA+20 diet was modified to contain 20% more branched-chain amino acids (BCAAs) than the Ctrl diet, while the BCAA−20 diet contained 20% fewer BCAAs. To ensure equal nitrogen content across all formulations, the Ctrl and BCAA−20 diets were supplemented with L-alanine to match the total nitrogen level of the BCAA+20 diet. The detailed ingredients and chemical compositions of the diets are found in Supplementary Table S1.

**Figure 1:**
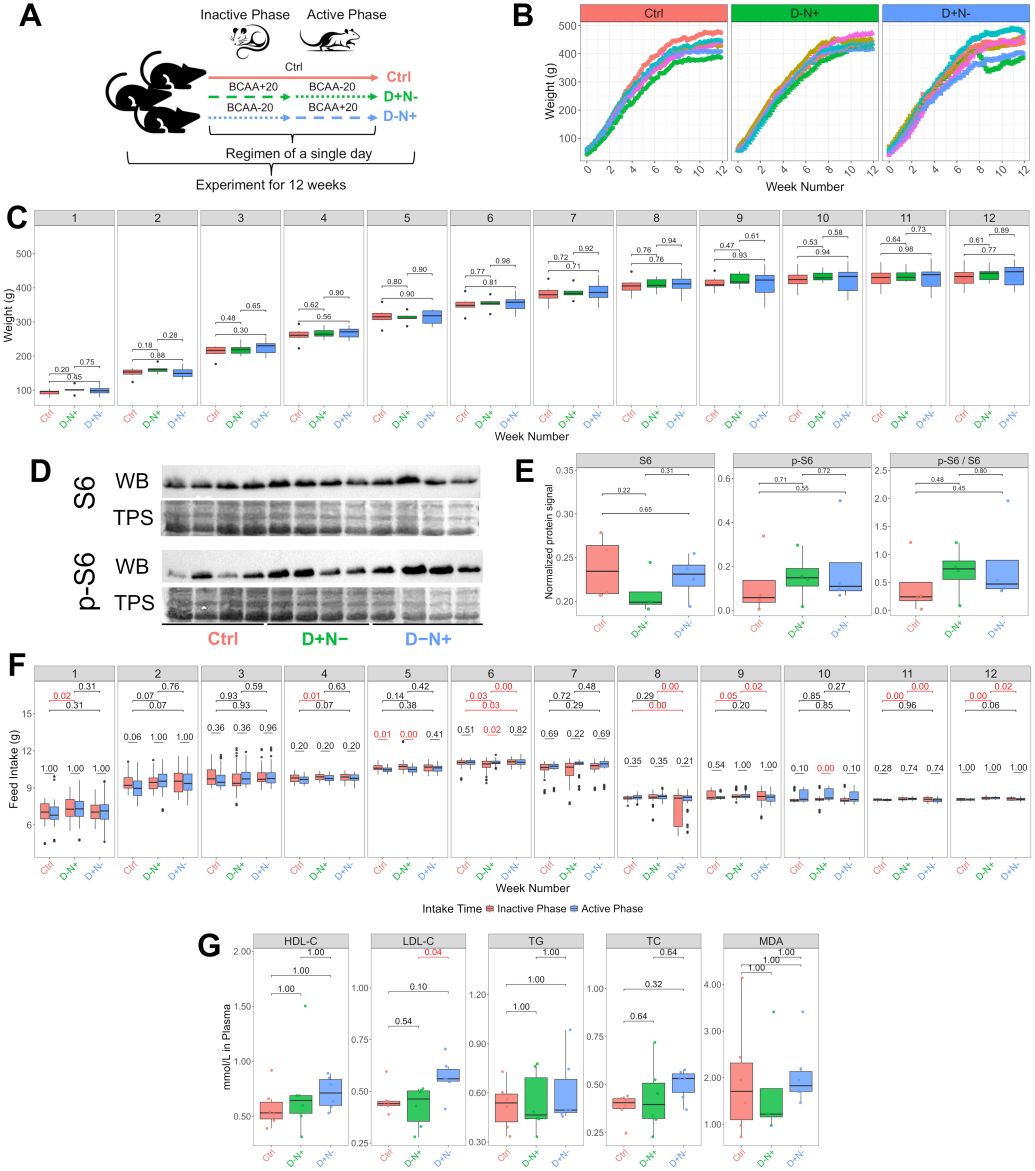
Experimental design, growth, intake partitioning, skeletal-muscle S6 signaling, and endpoint plasma markers under phase-shifted BCAA regimens. **(A)** Simplified study design: rats were maintained on a 12-h light:12-h dark cycle and assigned for 12 weeks to Ctrl (AIN-93G throughout), D + N − (BCAA+20 during the light/inactive phase and BCAA−20 during the dark/active phase), or D − N + (BCAA−20 during the light/inactive phase and BCAA+20 during the dark/active phase), where BCAA+20 and BCAA−20 denote diets containing ~20% higher or lower total BCAA content than Ctrl, respectively; daily energy and nitrogen were intended to be matched while allowing minor day-to-day variation during implementation (*n* = 6/group). **(B,C)** Individual body weight trajectories from week 0 (acclimation) to weeks 1–12 (experimental period) **(B)** and weekly body weight distributions shown as boxplots (weeks 1–12) with pairwise between-group comparisons annotated above brackets **(C)**. **(D,E)** Representative immunoblots of total ribosomal protein S6 and phosphorylated S6 (p-S6, Ser235/236) in skeletal muscle at the end of the study, with total protein staining (TPS; Ponceau S) used for normalization (*n* = 4/group; region approximately ~32 kDa displayed) **(D)**, and densitometric quantification of S6/TPS, p-S6/TPS, and phosphorylation ratio (p-S6/S6 computed as (p-S6/TPS)/(S6/TPS)) with individual animals overlaid **(E)**. **(F)** Weekly feed intake partitioned by circadian phase (inactive/light phase: 07:00–19:00; active/dark phase: 19:00–07:00); within each week, upper brackets/*p*-values compare whole-day (24 h) total intake between the groups, and lower brackets/*p*-values compare inactive *vs.* active intake within each group (red p-values indicate *p* < 0.05 as displayed). **(G)** Endpoint fasting plasma HDL-C, LDL-C, triglycerides (TG), total cholesterol (TC), and malondialdehyde (MDA) across the groups (units as indicated on axes). Group colors follow Ctrl (red), D − N + (green), and D + N − (blue). Boxplots show median and interquartile range (IQR), whiskers extend to 1.5 × IQR, and points indicate individual values (with outliers beyond whiskers where shown). Pairwise statistics were computed using two-sided t-tests with Holm *p*-value adjustment.

The simplified version of the experimental design is shown in Figure 1A. A total of 18 male Sprague–Dawley rats (age = 3 weeks, weight = 56.1 ± 7.15 g) were purchased from the Laboratory Animal Center of Guizhou Medical University and were entrained to a 12-h light:12-h dark cycle with *ad libitum* access to a standard diet for 3 days of acclimation. Then, animals were trained to consume the same amount of feed between light and dark phases for 7 days before the specific dietary intervention began. At the beginning of the experiment, animals were randomly assigned to three different dietary groups (*n* = 6 in each group), which are as follows: (1) Ctrl: a Ctrl diet throughout the whole day; (2) D + N−: BCAA+20 diet during 12-h inactive phase and BCAA−20 diet during the 12-h active phase; and (3) D − N+: BCAA−20 diet during the 12-h inactive phase and BCAA+20 diet during the 12-h active phase. Animals were fed according to the specific dietary program for 12 weeks, with free access to water.

At the end of the 12-week experimental period, the rats were fasted at the beginning of the active phase for 12 h. Subsequently, the animals were deeply anesthetized using 5% isoflurane inhalation. Once deep anesthesia was confirmed (loss of righting reflex and pain response), whole blood samples were collected via cardiac puncture until death. After confirming cessation of breathing and heartbeat and pupillary dilation, isoflurane administration was discontinued.

### 2.2 Untargeted metabolomics

#### 2.2.1 Sample preparation

Liver and skeletal muscle (quadriceps) samples were first weighed, then dried, and lyophilized. The dried samples were subsequently ground in a 2-mL Eppendorf tube containing a 5-mm tungsten bead for 1 min at 65 Hz using a grinding mill. Metabolites were extracted by adding 1 mL of a precooled mixture of methanol, acetonitrile, and water (v/v/v, 2:2:1) to each sample, followed by ultrasonic shaking for 1 h in an ice bath. Thereafter, the mixtures were stored at −20 °C for 1 h and centrifuged at 14,000 g for 20 min at 4 °C. The supernatants were collected and concentrated to dryness under vacuum. To ensure data quality in metabolic profiling, quality control (QC) samples were prepared by pooling aliquots from all samples. These QC samples were processed and analyzed using the same procedure as the experimental samples within each batch. The dried extracts were reconstituted in 50% acetonitrile, filtered through a disposable 0.22-μm cellulose acetate filter, transferred into 2-mL high-performance liquid chromatography (HPLC) vials, and stored at −80 °C until analysis.

#### 2.2.2 UHPLC–MS/MS analysis

Metabolomic profiling was performed by Shanghai Bioprofile Technology Co., Ltd. using an ultra-high-performance liquid chromatography (UPLC)-ESI-Q-Orbitrap-MS system (Shimadzu Nexera X2 LC-30 AD; Shimadzu, Japan) coupled with a Q-Exactive Plus mass spectrometer (Thermo Scientific, San Jose, USA). For liquid chromatography separation, samples were injected onto an ACQUITY UPLC® HSS T3 column (2.1 × 100 mm, 1.8 μm; Waters, Milford, MA, USA) with a flow rate of 0.3 mL/min. The mobile phase consisted of solvent A (0.1% formic acid in water) and solvent B (100% acetonitrile). The gradient program was as follows: 0% solvent B for 2 min, linearly increased to 48% over 4 min, further increased to 100% over the subsequent 4 min and held for 2 min, and then decreased to 0% in 0.1 min, followed by a 3 min re-equilibration period. Electrospray ionization (ESI) was applied in both positive and negative modes for separate data acquisitions. The heated electrospray ionization (HESI) source parameters were as follows: spray voltage: 3.8 kV (positive) and 3.2 kV (negative); capillary temperature: 320 °C; sheath gas flow (nitrogen): 30 arb; auxiliary gas flow: 5 arb; probe heater temperature: 350 °C; and S-lens radio frequency (RF) Level: 50. Full mass spectrometry (MS) data were acquired over an m/z range of 70–1,050 Da with a resolution of 70,000 (at m/z 200), while tandem mass spectrometry (MS/MS) data were acquired at a resolution of 17,500 (at m/z 200). The maximum injection times were 100 ms for MS and 50 ms for MS/MS. An isolation window of 2 m/z was employed for MS^2^, and fragmentation was performed using stepped normalized collision energies of 20, 30, and 40.

#### 2.2.3 Data preprocessing and filtering

Raw MS data were processed using Mass Spectrometry-Data Independent Analysis software (MS-DIAL) for peak alignment, retention time correction, and peak area extraction. Metabolites were identified based on accurate mass (mass tolerance < 10 ppm) and MS/MS spectra (mass tolerance < 0.02 Da) by comparison against the Human Metabolome Database (HMDB), MassBank, other public databases, and an in-house metabolite standard library. Among the extracted ion features, only those variables exhibiting more than 50% non-zero measurement values in at least one group were retained for further analysis.

#### 2.2.4 KEGG enrichment analysis

To identify the perturbed biological pathways, differential metabolites were subjected to the Kyoto Encyclopedia of Genes and Genomes (KEGG) pathway enrichment analysis using the KEGG database with the Fisher’s exact test, and FDR correction for multiple testing was performed. Enriched KEGG pathways were nominally statistically significant at the *p* < 0.05 level.

### 2.3 Biomarker assays

Biomarker assays were performed using commercially available kits from Elabscience (Wuhan, China) following the manufacturers’ protocols. Specifically, serum lipids were quantified by measuring total cholesterol (TC) using the Total Cholesterol (TC) Colorimetric Assay kits from Elabscience (Wuhan, China) (catalog no. E-BC-K109-M), high-density lipoprotein cholesterol (HDL-C) using the HDL-C Colorimetric Assay kits from Elabscience (Wuhan, China) (catalog no. E-BC-K221-M), low-density lipoprotein cholesterol (LDL-C) using the LDL-C Colorimetric Assay kits from Elabscience (Wuhan, China) (catalog no. E-BC-K205-M), malondialdehyde (MDA) using the Malondialdehyde (MDA) Colorimetric Assay kits from Elabscience (Wuhan, China) (catalog no. E-BC-K025-M), and triglycerides (TGs) using the Triglyceride (TG) Colorimetric Assay kits from Elabscience (Wuhan, China) (catalog no. E-BC-K261-M). Additionally, total superoxide dismutase (T-SOD) activity was measured using the T-SOD Activity Assay kits from Elabscience (Wuhan, China) (catalog no. E-BC-K019-M), and total/oxidized glutathione was assessed using the Total Glutathione (T-GSH)/Oxidized Glutathione (GSSG) Colorimetric Assay kits from Elabscience (Wuhan, China) (catalog no. E-BC-K097-M).

### 2.4 Western blot analysis

Skeletal muscle tissue samples were homogenized in radioimmunoprecipitation assay (RIPA) buffer supplemented with a protease inhibitor cocktail (1:100). The resulting lysates were then centrifuged at 16,000 × g for 20 min at 4 °C. The protein concentration of the supernatant was determined using a bicinchoninic acid (BCA) protein assay kit.

In the Western blot analysis, 15 μg of muscle extract per sample was subjected to sodium dodecyl sulfate–polyacrylamide gel electrophoresis (SDS-PAGE) on a 12% polyacrylamide gel at 120 V for approximately 80 min and subsequently transferred to a polyvinylidene difluoride (PVDF) membrane. After transfer, the membrane was stained with Ponceau S for total protein normalization.

The membranes were blocked for 1 h with 5% non-fat milk in Tris-buffered saline with 0.1% Tween 20 (TBS-T) and then immunoblotted overnight at 4 °C with primary antibodies (1:2000 in 5% non-fat milk) against S6 ribosomal protein (#2217) and phospho-S6 ribosomal protein (Ser235/236) (#4858). All antibodies were purchased from Cell Signaling Technology (Danvers, MA, USA). Following incubation with the primary antibodies, the membranes were washed three times with TBS-T and incubated for 1 h at room temperature with the appropriate horseradish peroxidase (HRP)-conjugated secondary antibodies. The dilution for the secondary antibodies was 1:5000 for S6 and 1:2000 for phospho-S6. Protein bands were visualized using an enhanced chemiluminescence (ECL) detection system, and bands were normalized by total protein density (total protein staining [TPS]) from Ponceau S.

### 2.5 Hematoxylin and eosin (H&E) staining

Liver and skeletal muscle tissues were fixed in 10% neutral buffered formalin for 24–48 h at room temperature, dehydrated through a graded ethanol series (70, 80, 95, and 100%), cleared in xylene, and embedded in paraffin wax. Sections of 4-μm thickness were cut using a rotary microtome and mounted on glass slides. After deparaffinization in xylene and rehydration through descending alcohol concentrations to distilled water, the sections were stained with hematoxylin for 5 min, rinsed in running tap water, differentiated briefly in 1% acid alcohol, and blued in alkaline water (0.2% ammonia). Slides were then counterstained with eosin Y for 1–2 min, dehydrated, cleared in xylene, and mounted with coverslips using a neutral resin. The stained sections were examined and scanned under a digital scanner (Pannoramic MIDI, Budapest, Hungary).

### 2.6 Statistical analysis

All data analyses and modeling were performed using R (version 4.0.3) and associated packages.

For physiological data, the statistical significance of differences between growth and intake curves was assessed using a permutation test implemented with the *compareTwoGrowthCurves()* function from the *statmod* package.

Growth uniformity was defined as the degree of inter-individual consistency in longitudinal body weight trajectories within each experimental group. Statistically, this was quantified by the magnitude of the within-group residual variance after accounting for fixed effects of time and group membership. Lower residual variance was interpreted as greater growth uniformity, indicating more homogeneous growth patterns among animals in the same group. To assess such growth uniformity, a linear mixed-effects model was fitted to the repeated body weight measurements using the *nlme* package. The model included day (time), group, and their interaction as fixed effects, with random intercepts and random slopes for time at the individual animal level to account for baseline differences and individual growth rates. To test whether growth uniformity differed between the groups, two nested models were compared: (1) a model assuming a common residual variance across all groups and (2) a model allowing group-specific residual variances. A likelihood ratio test was used to determine whether allowing heterogeneous residual variances significantly improved model fit, with statistical significance indicating differences in growth uniformity between the groups.

For metabolomic data, raw data were first scaled using Pareto scaling. Unsupervised principal component analysis (PCA) was used for initial visualization, followed by supervised orthogonal partial least-squares discriminant analysis (OPLS-DA) to identify discriminating features. The quality and predictive performance of the OPLS-DA models were evaluated using R^2^X, R^2^Y, and Q^2^ values and validated against overfitting with 200-iteration permutation tests.

Differentially expressed metabolites were identified using a multi-tiered approach. For valid orthogonal partial least squares-discriminant analysis (OPLS-DA) models, metabolites were considered significant if they met the criteria of both a variable importance in projection (VIP) score > 1.0 and a *p*-value of < 0.05. In cases where the OPLS-DA model permutation test indicated poor reliability, an alternative set of criteria was used: a fold change of ≥ 1.5 (or ≤ 1/1.5) and a p-value of < 0.05. *p-values* for group comparisons were calculated using two-tailed Student’s t-tests for pairwise comparisons with Holm-adjusted *p*-values or one-way analysis of variance (ANOVA) for comparisons involving more than two groups, performed on normalized data. Identified differential metabolites were subsequently used for cluster heat map analysis using the *ComplexHeatmap* package.

## 3 Results

### 3.1 Isocaloric BCAA regimens differentially impact growth uniformity and plasma lipid profiles

The regimens followed the experimental design as shown in Figure 1A. While all groups were targeted to receive the same total caloric load, we first assessed whole-animal physiological responses. The overall growth curves demonstrated a similar upward trajectory across all three groups (Figure 1B), and no body weight differences were observed throughout the experiment (Figure 1C). Markers of mTORC1-dependent translational signaling (ribosomal S6 and phosphorylated S6 protein expression) were measured in postmortem skeletal muscle samples, and no differences among groups were found (Figures 1D,E). Despite the non-significant differences in growth, Figure 1B shows distinct growth patterns among the groups, particularly after week 6. The Ctrl group exhibited a steady and progressive increase in body weight over time. In comparison, the D − N + group exhibited more uniform growth, whereas the D + N − group showed a more variable (turbulent) growth pattern, including one individual that experienced a period of stunted growth beginning at week 7 as the weight trend (green line in D + N−) started to decline but resumed to grow at week 9. To investigate whether there are differences in growth uniformity among the groups, we defined the growth uniformity as the within-group consistency of individual longitudinal body weight trajectories and statistically quantified it by residual variance after accounting for fixed effects of time and group using a linear mixed-effects model. It was revealed that the D − N + group, receiving supplemental BCAAs during the active phase, exhibited a significantly lower residual variance than the Ctrl and D + N − groups (L. Ratio = 9.067, *p* = 0.01), indicating more consistent and uniform growth. In contrast, the D + N − group exhibited greater variability, as shown and described.

Moreover, our investigation aimed to compare feeding regimens with redistributed BCAA content. Although the protocol targeted isocaloric and isonitrogenous conditions, significant differences in absolute whole-day intake persisted among the groups (Figure 1F; upper brackets), together with intermittent phase imbalances between the inactive and active periods (Figure 1F; brackets above the boxplots), most prominently in the D − N + group. However, after normalizing intake to body weight, these inter-group differences were abolished, and phase-related differences were no longer evident except for a single instance in D − N + at week 10 (Supplementary Figure S1).

**Figure 2:**
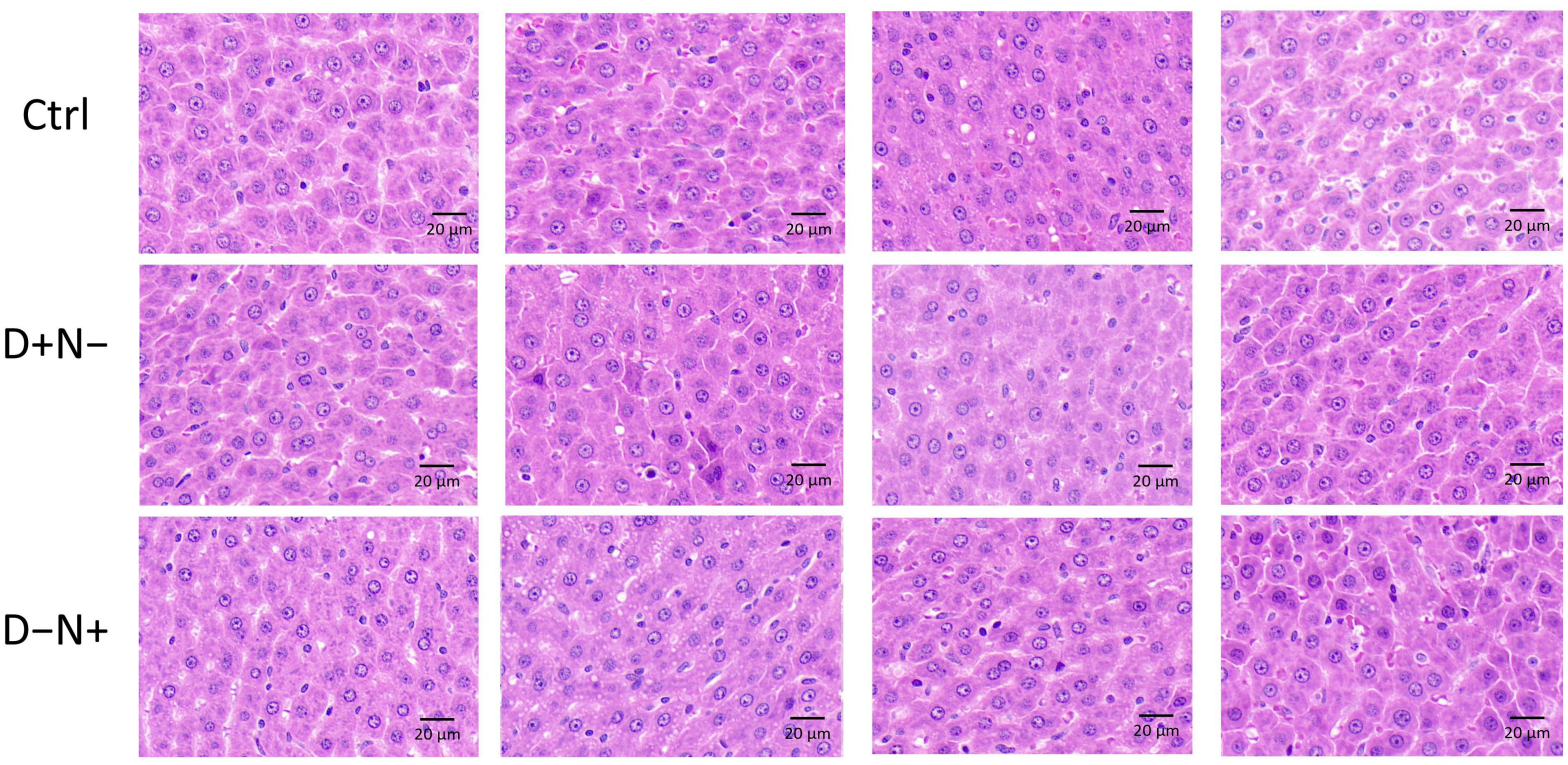
Representative H&E-stained sections of the livers from rats under different BCAA redistribution regimens. Liver histology from the control (Ctrl), BCAAs redistributed to the inactive phase (D + N−), and BCAAs redistributed to the active phase (D − N+) groups (four representative animals per group). Hepatocytes are arranged in normal hepatic cords with clear cytoplasm and centrally located nuclei. No apparent steatosis, necrosis, or inflammatory infiltration is observed among the groups. Scale bar = 20 μm.

**Figure 3:**
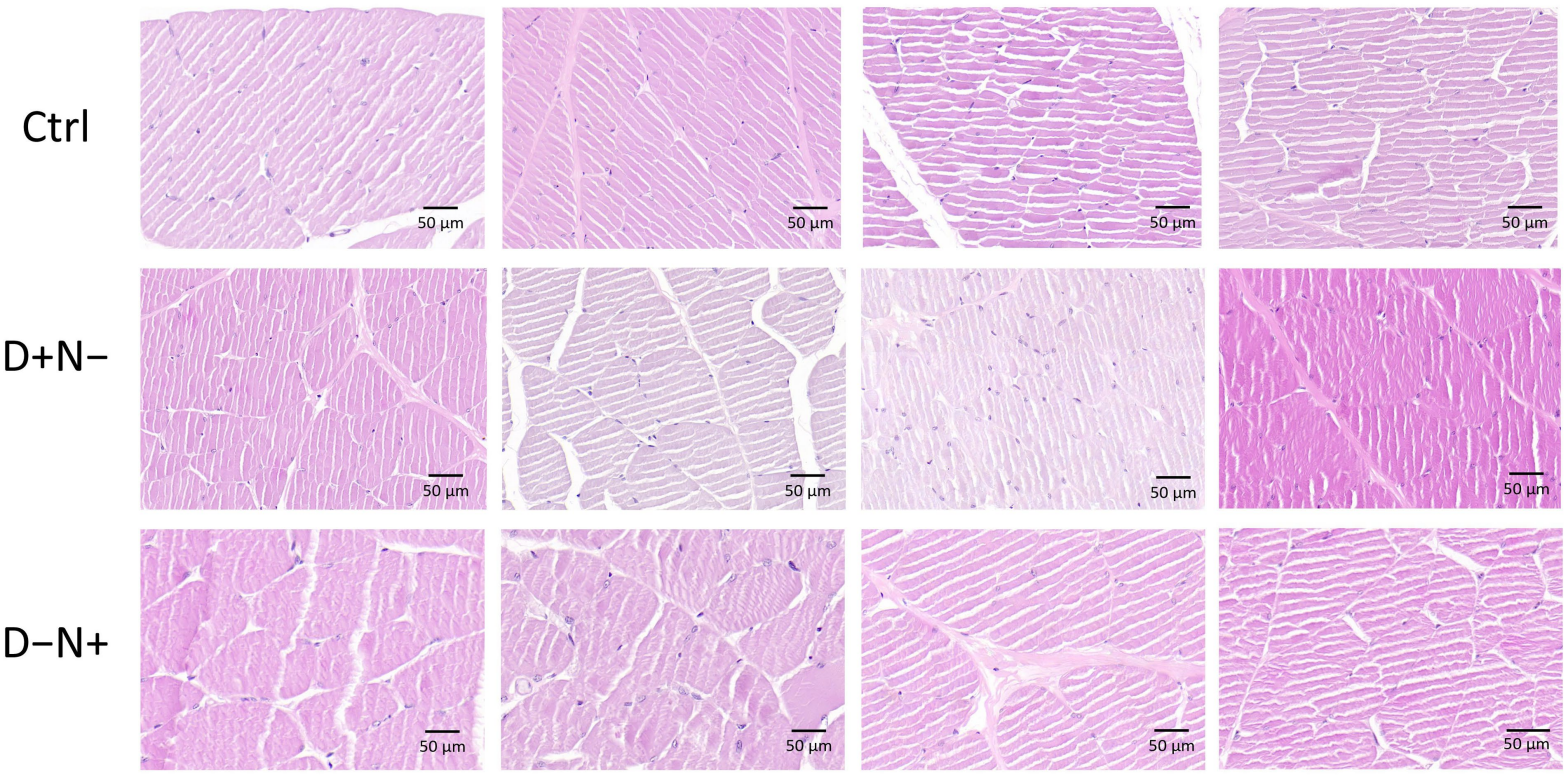
Representative H&E-stained sections of skeletal muscle from rats under different BCAA redistribution regimens. Skeletal muscle morphology from the control (Ctrl), BCAAs redistributed to the inactive phase (D + N−), and BCAAs redistributed to the active phase (D − N+) groups is shown (four representative animals per group). Muscle fibers display normal polygonal architecture with peripheral nuclei and no visible signs of myofibrillar degeneration, necrosis, or inflammatory infiltration across all groups. Scale bar = 50 μm.

Regarding systemic metabolic markers, plasma lipid analysis revealed a significant elevation of LDL-C (low-density lipoprotein cholesterol) in the D + N − group compared to the Ctrl group (*p* = 0.04) (Figure 1G). However, this shift was not accompanied by overt systemic toxicity. Histological evaluation of H&E-stained liver sections showed no visible pathological lesions (Figure 2), and no morphological differences were found in the skeletal muscle (Figure 3). Given these results, we proceeded to explore whether subtle metabolic shifts could underlie the observed lipid differences through untargeted metabolomics.

### 3.2 Metabolomic analysis reveals lipid metabolism as the primary driver of tissue-specific separation

**Figure 4:**
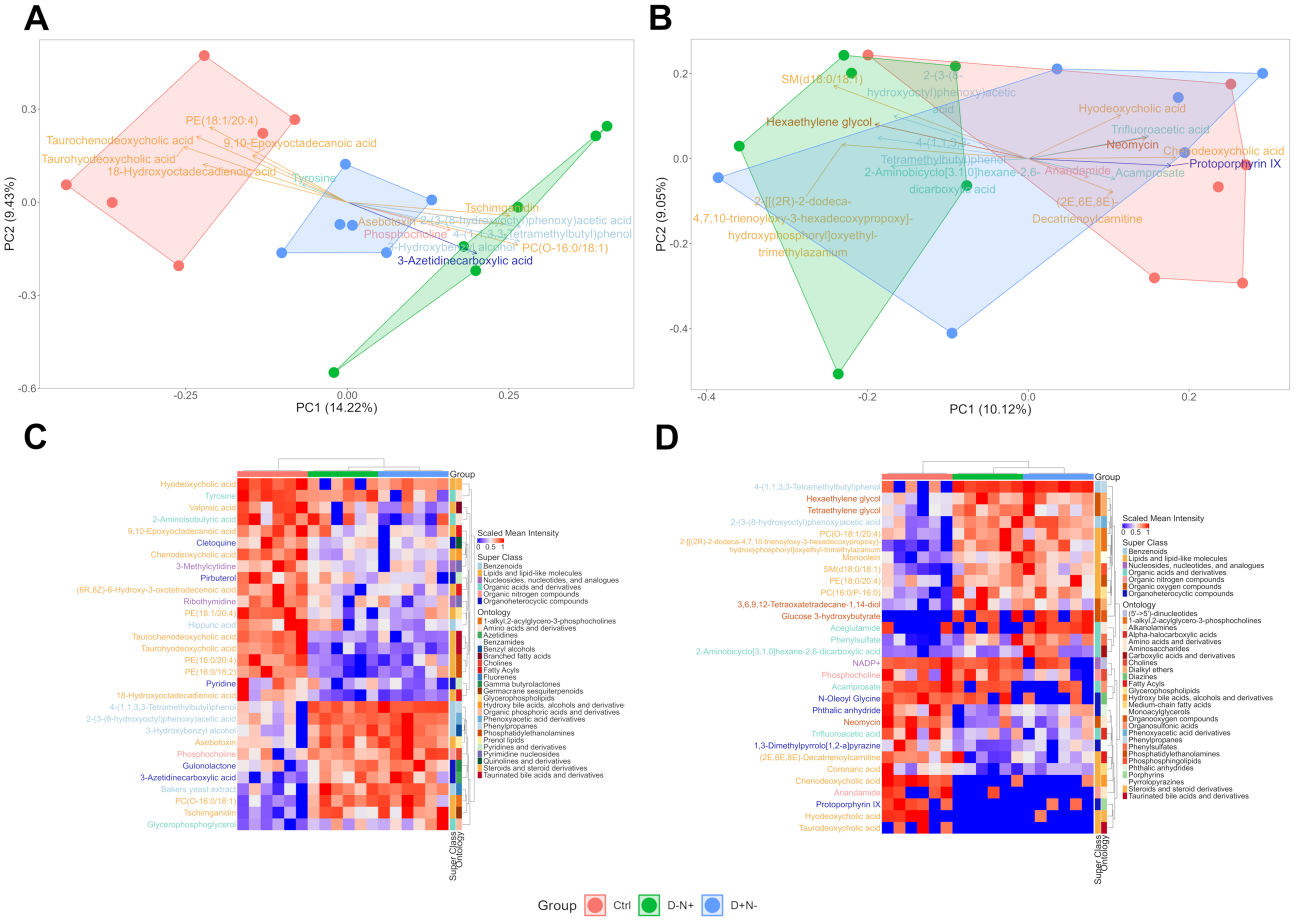
Principal component analysis (PCA) and hierarchical clustering of untargeted metabolomic profiles in the liver and skeletal muscle. **(A,B)** PCA score plots for liver **(A)** and skeletal muscle **(B)**; each point represents an individual sample, and shaded polygons denote group convex hulls (Ctrl, red; D − N+, green; D + N−, blue). Arrows indicate the top 15 loading vectors, with loading labels colored by metabolite superclass. **(C,D)** Hierarchical clustering heatmaps of the top 30 significantly different metabolites in the liver **(C)** and skeletal muscle **(D)**. Heatmap colors represent scaled mean intensity (0–1, as indicated). The top annotation bar denotes the experimental group, and side annotation bars indicate metabolite superclass and ontology according to the legend. Significantly different metabolites were defined by the intersection of univariate testing (*p* < 0.05) and fold-change filtering (FC > 1.5).

Untargeted metabolomics were performed on liver and skeletal muscle tissues. Principal component analysis (PCA) revealed distinct metabolic profiles across experimental groups, with the most robust separation occurring in the liver (Figures 4A,B). In both tissues, the control (Ctrl) and D − N + groups exhibited the greatest divergence, while the D + N − group occupied an intermediate metabolic space. Variable importance on projection (VIP) scores identified lipids and lipid-like molecules as the primary drivers of this separation.

Heatmap visualization of the top discriminating features further supported a lipid-centered shift in both tissues (Figures 4C,D). In the liver, several lipid subclasses contributed to the separation, including multiple bile acid-annotated features that were relatively higher in the Ctrl regimen and lower in both shifted BCAA regimens (D + N − and D − N+). Other lipid-related features (e.g., choline-related metabolites and glycerophospholipid-associated signals) exhibited more heterogeneous patterns across the groups. In skeletal muscle, although the overall separation was weaker in PCA, the top discriminating features encompassed a broader mix of chemical ontologies (lipid and non-lipid annotations), giving the displayed profile a more “diverse” appearance.

The original aligned and annotated data matrix are found in Supplementary Table S2.

### 3.3 BCAA-related metabolites are largely unchanged across the experimental groups

**Figure 5:**
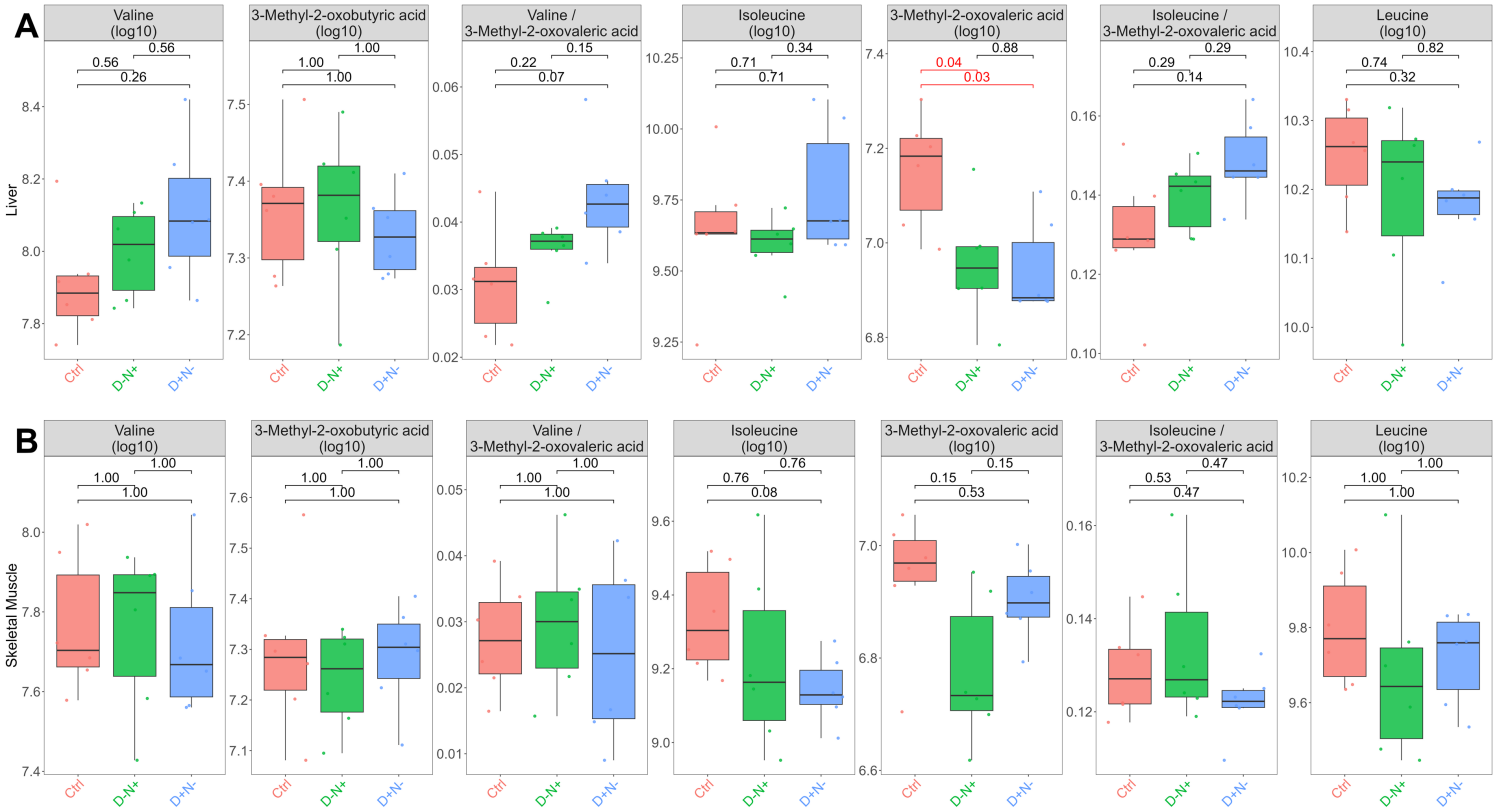
Distributions of branched-chain amino acids (BCAAs), detected branched-chain keto acids (BCKAs), and BCAA-to-BCKA ratios in the liver **(A)** and skeletal muscle **(B)** from untargeted metabolomic features (log10 scale). Ketoleucine (4-methyl-2-oxopentanoate) was not detected. Brackets annotate pairwise between-group comparisons; values shown are Holm-adjusted *p*-values from two-sided t-tests, with red indicating adjusted *p-values of* < 0.05. Groups are colored as Ctrl (red), D − N + (green), and D + N − (blue). Boxplots show the median and interquartile range (IQR), whiskers extend to 1.5 × IQR; and points indicate individual observations (with outliers beyond whiskers shown where present).

Given the dietary intervention, we quantified the peak intensities of BCAAs and their primary catabolites, branched-chain keto acids (BCKAs), in both tissues. The leucine flux ratio could not be calculated because leucine’s corresponding BCKA, 4-methyl-2-oxopentanoate, was undetectable. In the liver (Figure 5A), valine, isoleucine, leucine, and most detected BCKAs did not differ among the groups. However, 3-methyl-2-oxovaleric acid differed modestly between the groups, with lower levels in the D − N + group compared with the Ctrl (*p* = 0.04) and D + N − (*p* = 0.03) groups. In the skeletal muscle (Figure 5B), neither BCAAs nor detected BCKAs differed significantly among the groups.

### 3.4 Timing-dependent BCAA intake is associated with hepatic redox homeostasis and antioxidant defense, while skeletal muscle shows enhanced anabolic programming

**Figure 6:**
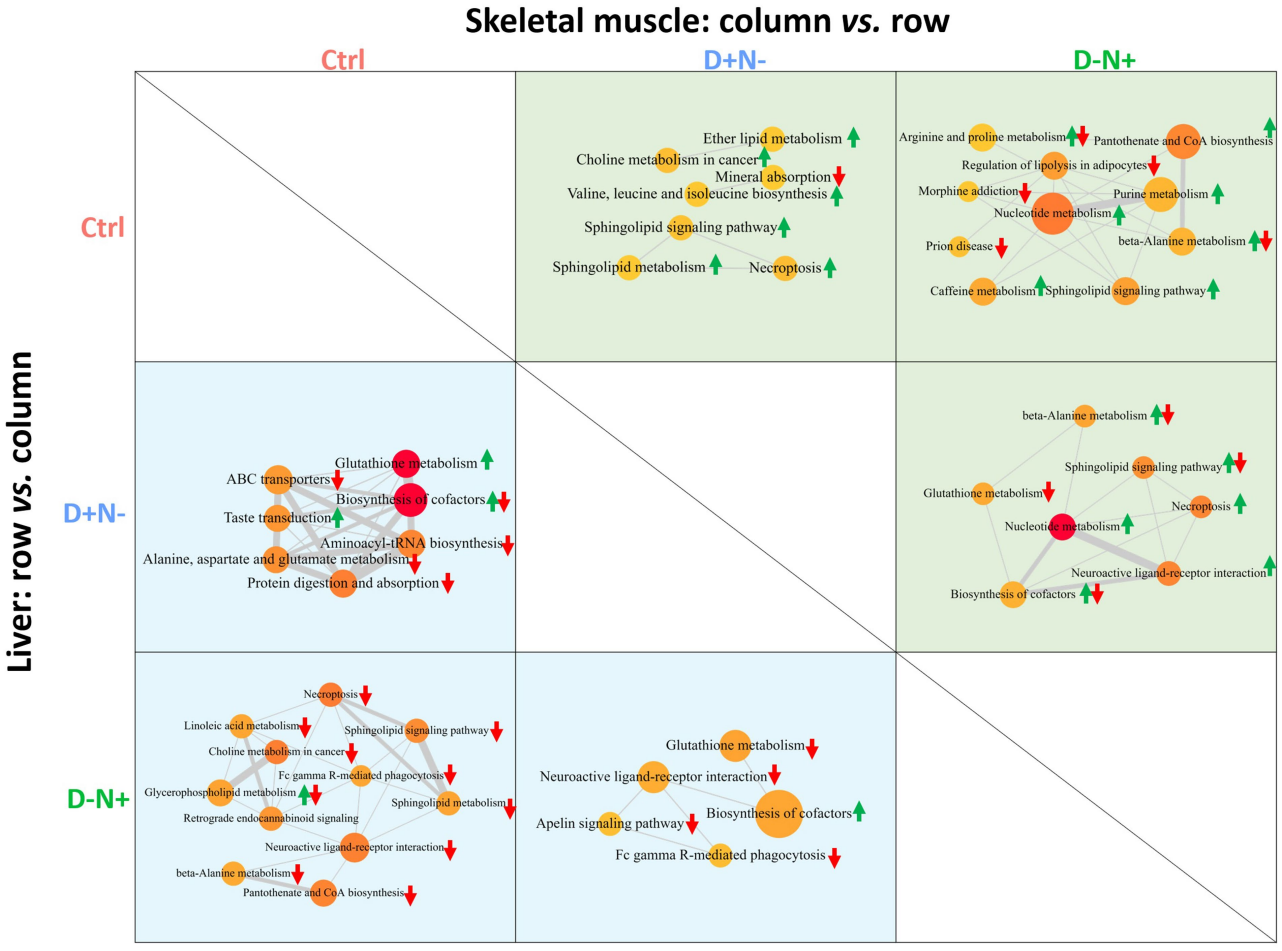
Pathway–pathway interaction analysis reveals tissue-specific metabolic signatures driven by BCAA timing in the liver and skeletal muscle. The grid illustrates the functional analysis of differentially expressed metabolites, comparing the Ctrl, D + N−, and D − N + groups. The analysis is separated by tissue: the liver is shown in the bottom-left triangle (blue background), and the skeletal muscle is in the top-right triangle (green background). For the liver, comparisons are made between the group in the row and the group in the column (row *vs.* column). For the skeletal muscle, the comparison is reversed (column *vs.* row). Each circular node represents a metabolic pathway, with the node size being proportional to the number of identified metabolites involved. The statistical significance (*p*-value) of the pathway enrichment is represented by color, where a more intense red signifies a lower *p*-value. The overall regulation of each pathway is indicated by arrows: a green up-arrow for upregulation, a red down-arrow for downregulation, and both arrows for mixed regulation.

To contextualize the metabolic alterations induced by nutrient timing, a pathway–pathway interaction analysis was performed, revealing distinct hepatic and muscular signatures governed by the phase of BCAA intake (Figure 6).

In the liver, compared with the Ctrl, the D + N − group exhibited the upregulation of glutathione metabolism and the downregulation of protein digestion and absorption, along with alterations in amino acid and cofactor-related pathways. In contrast, the D − N + group showed the downregulation of necroptosis and sphingolipid signaling pathways compared to the D + N − and Ctrl groups, together with modulation of lipid-associated pathways.

Skeletal muscle exhibited a distinct response profile. Compared to Ctrl, D − N + was associated with enrichment of biosynthetic and energy-supporting pathways, including purine metabolism, nucleotide metabolism, and pantothenate and CoA biosynthesis, consistent with a shift toward increased anabolic capacity. In the D + N − versus D − N + comparison, muscle pathway changes were comparatively modest and frequently mixed-direction, with the most prominent signals involving nucleotide metabolism and neuroactive ligand–receptor interaction.

To corroborate the redox-related pathway signal in the liver, we quantified key antioxidant indices using colorimetric assays. Although GSH/GSSG did not differ significantly among the groups, T-SOD activity displayed a clear phase-dependent pattern: the D − N + group showed higher hepatic T-SOD activity than the D + N − group (711.91 *vs.* 619.86 U/mg protein; *p* < 0.05; Supplementary Figure S2).

## 4 Discussion

### 4.1 Practical constraints and stress-linked consequences of circadian BCAA scheduling

The study was designed to be strictly isocaloric and isonitrogenous across inactive and active phases, but this could not be maintained consistently without animal welfare concerns. Therefore, the regimen was implemented in a nominally isocaloric and isonitrogenous manner. The main constraints were reduced intake of the BCAA−20 diet and the naturally lower drive to eat during the inactive phase. This is consistent with amino acid-balancing behavior in rats, which prefer diets with balanced essential amino acids and avoid diets deficient in a single Essential amino acid (EAA) (15, 16), which made D − N + the most difficult condition to maintain. Accordingly, weeks 5, 6, and 10 showed significant active–inactive intake imbalance (Figure 1F), likely because active-phase BCAA+20 intake reduced subsequent inactive-phase intake of BCAA−20.

In contrast, D + N − was easier to implement because its active–inactive intake ratio was more stable. However, total daily intake differed from other groups at several time points (Figure 1F). Thus, the main deviation in D + N − was not only when the animals ate but also how much they ate on certain days. In addition, the circadian misalignment in D + N − —high energy demand during the active phase combined with BCAA deprivation—may have increased physiological strain. This may explain the more variable growth pattern, including the marked attenuation in one animal as shown in Figure 1B. Such instability is compatible with stress-eating and allostatic load models, in which repeated, partly uncontrollable challenges disturb feeding regulation (17).

Since absolute intake is closely related to body weight, we also analyzed intake normalized to body weight (Supplementary Figure S1). After normalization, most inter-group and inter-phase differences were largely abolished. Skeletal muscle p-S6/S6 also did not differ among the groups (Figures 1D,E). Since all animals were sampled at the same circadian time, this lack of difference is unlikely to be caused by phase mismatch. Instead, it suggests that redistributing BCAA intake alone did not produce a sustained change in muscle mTORC1 signaling at terminal active-phase sampling. This interpretation is supported by prior studies showing that p-mTOR/p-S6 responses after EAA/protein feeding (18, 19) or resistance training (20) are transient and return to baseline within hours. A previous study further showed that BCAAs can promote cardiac growth at the end of the active phase through BMAL1-dependent, time-of-day-specific mTOR activation (11, 21). Taken together, our data suggest that under near-isocaloric/isonitrogenous conditions, absolute BCAA dose rather than inactive/active redistribution was the main determinant of sustained skeletal muscle mTORC1 readouts.

### 4.2 Inactive-phase BCAA enrichment coincides with LDL-C elevation, while hepatic lipid-feature shifts are shared across shifted regimens

Although the overall growth differences were modest, D + N − showed a more variable growth trajectory together with elevated plasma LDL-C (Figure 1G). Since the current design cannot disentangle whether this phenotype reflects reduced BCAA availability during the active phase versus excess BCAA exposure during the inactive phase, we interpret the LDL-C change in the context of prior evidence. Human studies reported associations between higher BCAA intake and higher LDL-C (22, 23), and mice fed a BCAA-deficient diet exhibit lower LDL-C (24). Chrononutrition studies further indicated that nutrient intake during the rest phase predisposes to dyslipidemia, whereas feeding aligned with the active phase favors lipid oxidation and metabolic flexibility (8, 25, 26). In our study, since (i) Ctrl and D + N − differed only modestly in the active–inactive intake ratio and (ii) whole-day intake differed only slightly between the groups, the LDL-C elevation in D + N − is more consistent with timing-dependent disproportionate BCAA exposure.

Since H&E staining showed no obvious liver pathology (Figure 2), we used metabolomics to detect subtler tissue responses. In the liver, the dominant separation was lipid-led and included several putative bile acid-related features, including taurine-conjugated species, which were lower in both shifted BCAA groups than in the Ctrl group. Since bile acid pools and enterohepatic cycling are highly sensitive to feeding time and circadian context, these signals may indicate altered hepatic lipid handling and/or gut–liver metabolic coupling. However, because the annotations were untargeted and bile acids were not directly quantified, this finding should be treated as hypothesis-generating rather than mechanistic evidence.

### 4.3 Feature-level metabolomics suggests heterogeneous, lipid-led muscle remodeling without an endpoint BCAA pool or mTORC1 shift

In the skeletal muscle, the metabolomic separation was likewise lipid-led, with membrane-associated lipid/phosphocholine features elevated in both shifted BCAA groups and relatively higher putative bile acid−/carnitine-related features in the Ctrl group. However, many of the top-ranking compounds lacked clear biological annotation (Figure 4D) and did not cleanly discriminate experimental groups on PCA (Figure 4B), indicating that feature-level changes in muscle were more heterogeneous and harder to interpret mechanistically. Moreover, BCAAs and detected BCKAs were generally unchanged (Figure 5). Together, these observations suggest that, under the terminal sampling condition, skeletal muscle is unlikely to be a primary endpoint tissue for sustained shifts in the BCAA/BCKA pool; instead, it may reflect secondary remodeling of lipid handling downstream of the systemic (liver-centered) phenotype (27).

This interpretation is biologically plausible. Quadriceps avidly takes up BCAA and is a major site of the first step of BCAA catabolism (3, 28), but pool sizes may still remain stable because the animals were closer to a postabsorptive state at sacrifice and because BCAA handling is highly flux-driven. Thus, unchanged abundance does not rule out altered turnover. Consistently, the absence of group differences in p-S6/S6 argues against a sustained difference in muscle mTORC1 tone at end-of-active-phase sampling; leucine/BCAA activation of mTOR signaling is typically transient (29). We therefore moved from feature-level analysis to pathway-level integration to determine whether coordinated biological programs emerged from these distributed changes (Figure 6).

### 4.4 Pathway networks implicate hepatic stress/redox responses and muscle metabolic remodeling under BCAA timing shifts

Figure 6 consolidates the metabolomics-derived pathway enrichment into a tissue-resolved, direction-aware interaction network, and it clarifies the logic behind the phenotypes observed earlier: inactive-phase BCAA enrichment (D + N−) preferentially engages hepatic stress/nutrient-handling programs, whereas active-phase enrichment (D − N+) shifts the system toward a normal or lower-stress hepatic state and a more biosynthetically “supported” muscle state.

In the liver, the D + N − *vs.* Ctrl comparison is dominated by upregulated glutathione metabolism alongside coordinated downregulation of protein digestion/absorption and amino acid-handling modules (e.g., aminoacyl-tRNA biosynthesis and alanine/aspartate/glutamate metabolism). Functionally, this pattern is most consistent with a compensatory redox-response signature occurring in parallel with impaired or inefficient nutrient processing. The liver appears to be allocating capacity toward antioxidant/defense-associated metabolism (30) while showing a relative contraction in pathways that report on amino acid availability and utilization (31). This is coherent with the behaviorally unstable growth trajectory and the LDL-C elevation observed, suggesting that the dominant signal is metabolic strain rather than frank tissue damage, even in the absence of overt histologic injury. By contrast, the D − N + liver network is characterized by broad downregulation of pathways that commonly accompany inflammatory or stress signaling, including necroptosis, Fc gamma receptor (FcγR)-mediated phagocytosis, and sphingolipid signaling/metabolism. This pattern is directionally consistent with reports that aligning nutrient intake to the active phase and/or applying time-restricted feeding can reduce hepatic inflammatory/stress tone across experimental and clinical settings (10, 32, 33). This “lower danger tone” is also directionally concordant with our biochemical validation: hepatic T-SOD activity is higher in D − N + than D + N − (Supplementary Figure S2), implying stronger inducible antioxidant defense when BCAA availability is aligned with the active phase.

In the skeletal muscle, the D − N + *vs.* Ctrl comparison reveals a coordinated upregulation of purine metabolism, nucleotide metabolism, and pantothenate/CoA biosynthesis. This metabolic “triad” likely enhances ATP turnover, nucleotide supply, and acyl-CoA-dependent flux, creating a cellular environment optimized for biosynthetic and mitochondrial work. In stark contrast, the D + N − *vs.* Ctrl group exhibits a shift toward necroptosis and sphingolipid-related signaling—a signature indicative of cellular stress and repair rather than a sustained anabolic program. Mechanistically, sphingolipids such as ceramides and Sphingosine-1-Phosphate (S1P) act as critical signaling nodes that link nutrient oversupply to insulin resistance and inflammatory pathways (34). Critically, these divergent pathway shifts occur despite muscle BCAA/BCKA pool sizes and p-S6/S6 ratios remaining unchanged across the groups. This leads to a parsimonious conclusion: the redistribution of BCAA across the light–dark cycle does not result in a sustained change in terminal mTORC1 tone. Instead, the observed muscle signature reflects a remodeling of downstream substrates and energy metabolism rather than a persistent, BCAA-driven translational response.

### 4.5 Limitations and future directions

Several limitations should be acknowledged. First, we did not directly measure key regulators of BCAA catabolism, including Branched-Chain-Keto Acid Dehydrogenase (BCKDH) activity/phosphorylation and its modulators Branched-Chain-Ketoacid Dehydrogenase Kinase (BDK)/Protein Phosphatase, Mg2+/Mn2+ Dependent 1K (PPM1K), nor did we assess time-of-day-resolved clock outputs. Second, although the diet was designed to be isocaloric and isonitrogenous, unavoidable variation in total and phase-specific intake may confound timing effects. Third, the sample size was relatively small, which reduced power and increased susceptibility to inter-individual variability. Fourth, practical constraints prevented frequent fasting and serial blood sampling; therefore, we could not track longitudinal glucose, insulin, or related metabolic indices; define an objective physiological endpoint during the intervention; or monitor real-time metabolic status. Finally, we did not measure whole-body energy expenditure or spontaneous activity, so changes in behavior or expenditure may have contributed to the lipid and metabolomic phenotypes.

Future studies should combine direct assessment of the BCKDH regulatory axis with circadian sampling, ideally using isotope-tracer flux approaches. They should also improve intake matching or use pair-feeding, increase sample size, and include longitudinal monitoring of glucose/insulin dynamics, indirect calorimetry, and activity. Euthanasia at multiple circadian time points will be important to connect timing-dependent molecular signatures with whole-body physiology and clinically relevant outcomes.

## 5 Conclusion

In this preliminary study, redistributing BCAA intake across the light–dark cycle produced timing-dependent metabolic effects despite no differences in final body weight, overt histopathology, or sustained skeletal-muscle mTORC1 signaling. The liver showed the clearest response: inactive-phase BCAA enrichment (D + N−) was associated with higher plasma LDL-C and hepatic redox/stress-related remodeling, whereas active-phase enrichment (D − N+) was associated with more uniform growth, lower hepatic stress-related pathway enrichment, and higher hepatic T-SOD activity. Together, these findings identify the liver as the primary tissue responsive to BCAA timing and suggest that aligning BCAA enrichment with the active phase is metabolically more favorable than shifting it to the inactive phase.

## Data Availability

The original contributions presented in the study are included in the article/Supplementary material, further inquiries can be directed to the corresponding authors.
