# Supplementary material for: Redistribution of branched-chain amino acid intake between active and inactive phases modulates hepatic metabolism in rats
Source: Front Nutr. 2026 Apr 9;13:1754879. doi: 10.3389/fnut.2026.1754879 (PMC13102662; doi:10.3389/fnut.2026.1754879)
Supplement: Supplementary file 1 [file Supplementary_file_1.zip › Supplementary Figure S.PPTX]

## Slide 1
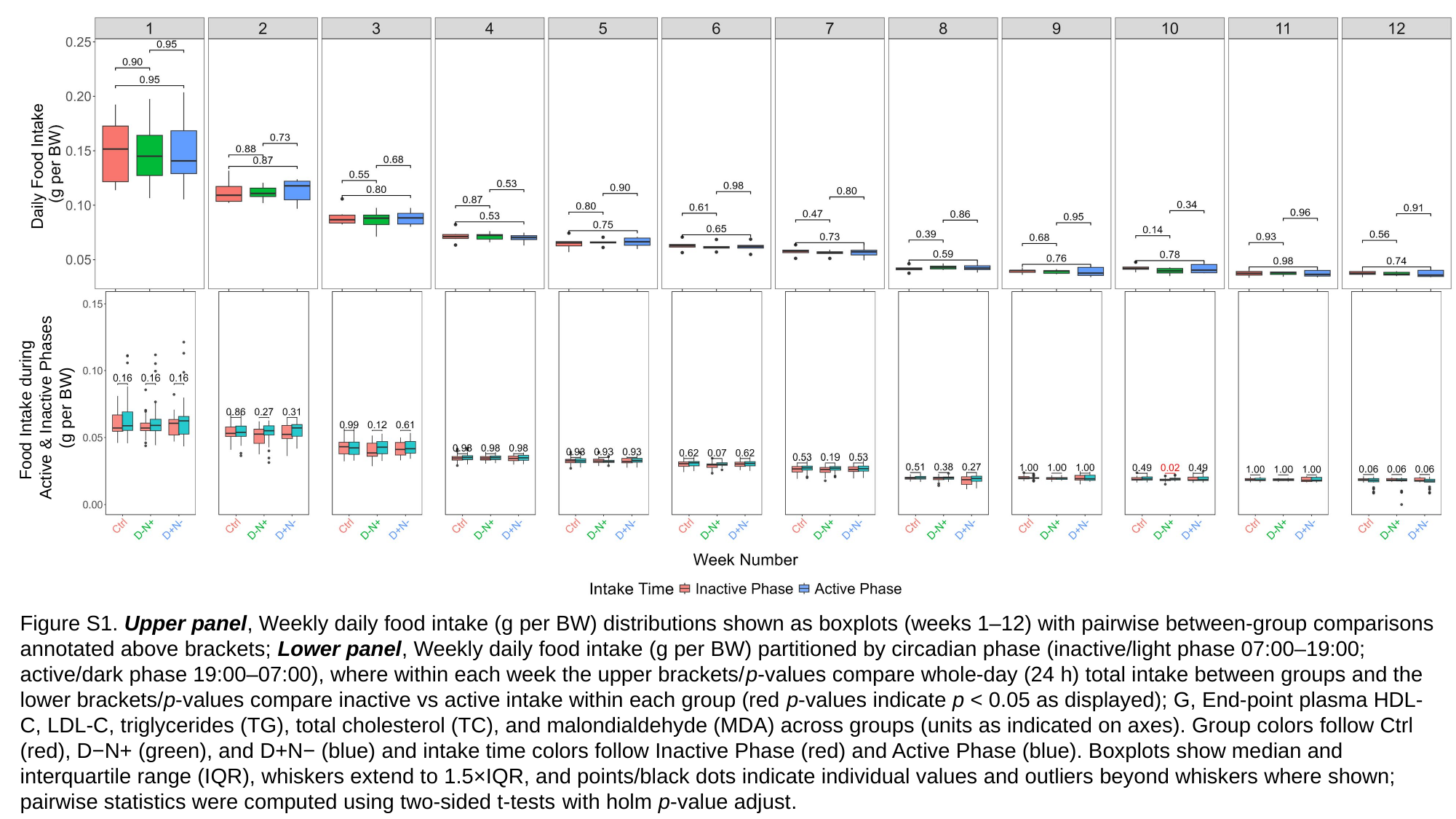

Food Intake during
Active & Inactive Phases
(g per BW)
Figure S1. Upper panel, Weekly daily food intake (g per BW) distributions shown as boxplots (weeks 1–12) with pairwise between-group comparisons annotated above brackets; Lower panel, Weekly daily food intake (g per BW) partitioned by circadian phase (inactive/light phase 07:00–19:00; active/dark phase 19:00–07:00), where within each week the upper brackets/p-values compare whole-day (24 h) total intake between groups and the lower brackets/p-values compare inactive vs active intake within each group (red p-values indicate p < 0.05 as displayed); G, End-point plasma HDL-C, LDL-C, triglycerides (TG), total cholesterol (TC), and malondialdehyde (MDA) across groups (units as indicated on axes). Group colors follow Ctrl (red), D−N+ (green), and D+N− (blue) and intake time colors follow Inactive Phase (red) and Active Phase (blue). Boxplots show median and interquartile range (IQR), whiskers extend to 1.5×IQR, and points/black dots indicate individual values and outliers beyond whiskers where shown; pairwise statistics were computed using two-sided t-tests with holm p-value adjust.

## Slide 2
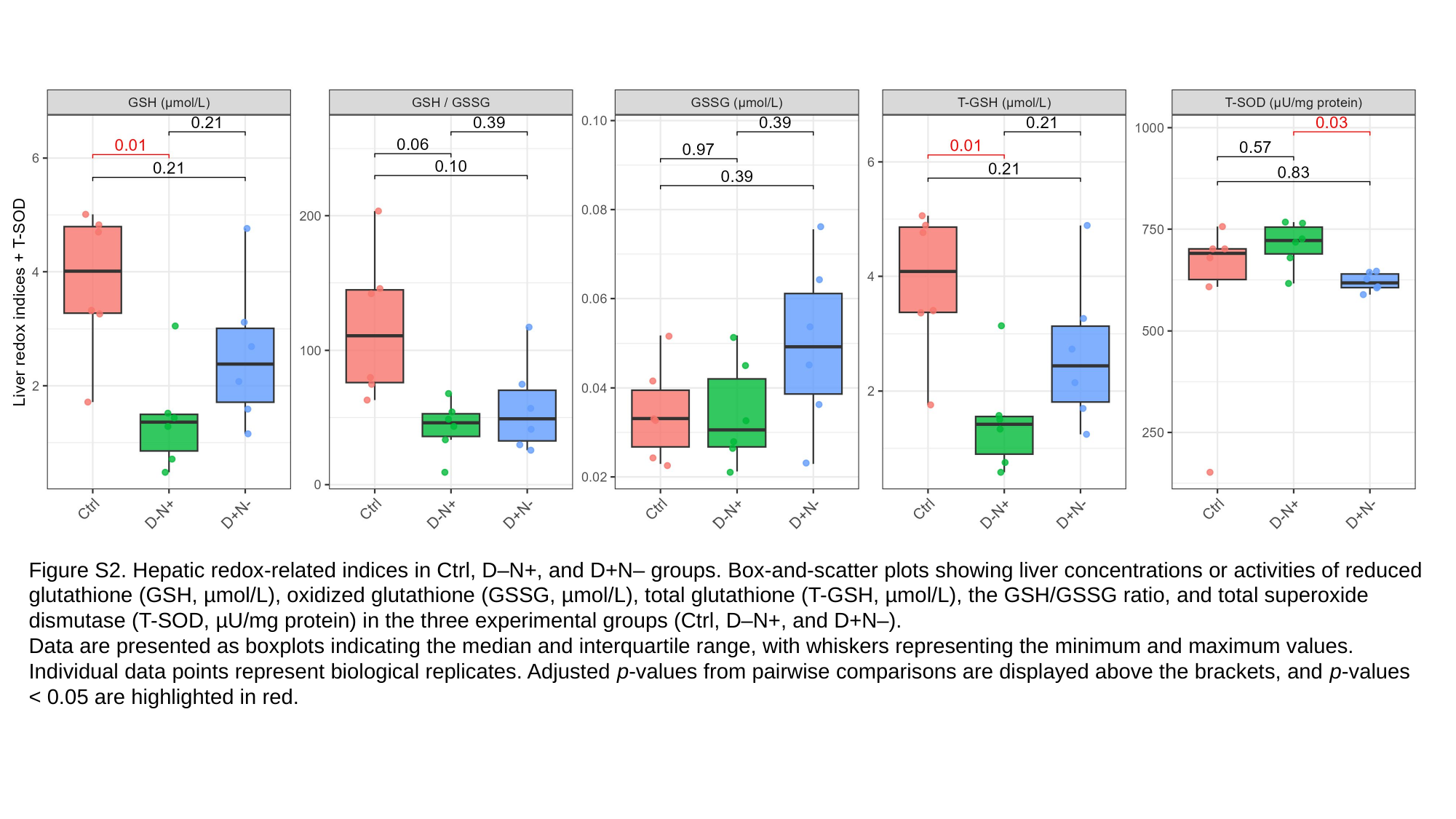

Figure S2. Hepatic redox-related indices in Ctrl, D–N+, and D+N– groups. Box-and-scatter plots showing liver concentrations or activities of reduced glutathione (GSH, µmol/L), oxidized glutathione (GSSG, µmol/L), total glutathione (T-GSH, µmol/L), the GSH/GSSG ratio, and total superoxide dismutase (T-SOD, µU/mg protein) in the three experimental groups (Ctrl, D–N+, and D+N–).
Data are presented as boxplots indicating the median and interquartile range, with whiskers representing the minimum and maximum values. Individual data points represent biological replicates. Adjusted p-values from pairwise comparisons are displayed above the brackets, and p-values < 0.05 are highlighted in red.
